# Ultrasound-Guided Epidural Anaesthesia in a 73-Year-Old Female With Bilateral Osteoarthritis and a History of Difficult Epidural Placement: A Case Report

**DOI:** 10.7759/cureus.70907

**Published:** 2024-10-05

**Authors:** Disheeta Bhalsod, Karuna Taksande, Amreesh Paul, Vatsal Patel

**Affiliations:** 1 Anaesthesiology, Jawaharlal Nehru Medical College, Datta Meghe Institute of Higher Education and Research, Wardha, IND; 2 Orthopedics, Jawaharlal Nehru Medical College, Datta Meghe Institute of Higher Education and Research, Wardha, IND

**Keywords:** difficult epidural placement, neuraxial anesthesia, osteoarthritis, total knee replacement, ultrasonography, ultrasound-guided epidural anaesthesia

## Abstract

Osteoarthritis (OA) of the knee is highly prevalent in the elderly population and generally causes disabling pain and dysfunction. Thus, knee OA has become one of the most common indications for total knee replacement (TKR) surgery. Neuraxial anaesthesia management in patients could be very challenging when there is a previous history of difficult epidural placements. The anatomical deformities and degenerative changes can limit the precise positioning of needles and increase the complication rates. This is a case of ultrasound-guided epidural and spinal anaesthesia administration in a 73-year-old female with a history of bilateral OA of the knee with previous difficult epidural placement. The patient had severe OA with severe pain and significant limitations in function. Epidural catheter placement was difficult in a prior bilateral tibial fracture repair, and she needed general anaesthesia for the procedure. Preoperative imaging showed significant degenerative change and decreased intervertebral spaces, which could compromise the accuracy of conventional epidural placement techniques. Real-time ultrasound was used for both epidural and spinal anaesthesia. Intraoperative and postoperative analgesia was provided with bolus doses of bupivacaine following the test dose that confirmed correct catheter placement. On the third postoperative day, the patient was discharged with oral analgesics. This report describes the use of ultrasound guidance to overcome the anatomical obstacles and challenges in epidural and spinal anaesthesia. The use of ultrasound guidance can contribute to a decrease in the complication rate, ensure adequate analgesia, and increase the accuracy of epidural treatment.

## Introduction

Osteoarthritis (OA) is a degenerative joint disorder that attacks the knees of elderly patients and is associated with extraordinary pain, stiffness, and loss of functional abilities. Cases with severities are most commonly treated by surgery, one of which is total knee replacement (TKR), relieving pain and improving such joint functioning [1]. In elderly patients with OA, the administration of anaesthesia has presented some intricate difficulties, particularly if prior difficulties inserting epidural catheters have occurred. Epidural anaesthesia is considered the standard of care for lower limb and intra-abdominal surgeries. It provides excellent analgesia and decreases the requirement for systemic analgesics. Although epidural anaesthesia offers a myriad of advantages, its ease of administration may be limited by degenerative changes as well as anatomic variations common in older patients with OA. Various factors may impede the right positioning of the catheter: distorted anatomical landmarks, reduced intervertebral spaces, and modified epidural structure [2].

Traditional approaches to epidural placements rely on palpation and anatomical reference points, which can be imprecise in cases with extensive degenerative changes or complications of previous interventions. Ultrasound guidance has perhaps become a significant advance for neuraxial anaesthesia in modern times. It provides the user with multiple advantages over conventional techniques. Real-time ultrasound visualization allows detailed imaging of the spinal anatomy. It thus permits more precise localization of the epidural space, further accuracy in needle placement, and a reduction in some of the complications like accidental dural puncture or inadequate block. These are useful in enhancing the visualization of anatomical structures that may become distorted or obscured as a result of degenerative changes and, by that very virtue, very helpful in increasing the success rate of neuraxial procedures [3]. Recent studies proved that ultrasound-guided epidural anaesthesia has a higher success rate with lower complication rates when compared to the traditional landmark-based approaches. It thus provides for the placement of the needle and the advancement of a catheter in patients whose anatomy is complicated to detect or with a history of neuraxial block placements that are problematic. It reduces the number of attempted insertions of the needle by lowering discomfort to the patients and any potential failure of the procedure through ultrasound guidance [4].

The current report presents a case of ultrasound-guided neuraxial anaesthesia in a 73-year-old female patient with a history of bilateral knee OA and prior difficult epidural insertions. We applied ultrasound guidance to overcome the anatomical challenges presented by the degenerative changes in this patient and complications related to previous anaesthetics. This concept ensured an optimal approach toward perioperative analgesia and patient safety by a dual mechanism, enabling appropriate placement of epidural catheters and providing a practical alternative to general anaesthesia.

## Case presentation

**Figure 1:**
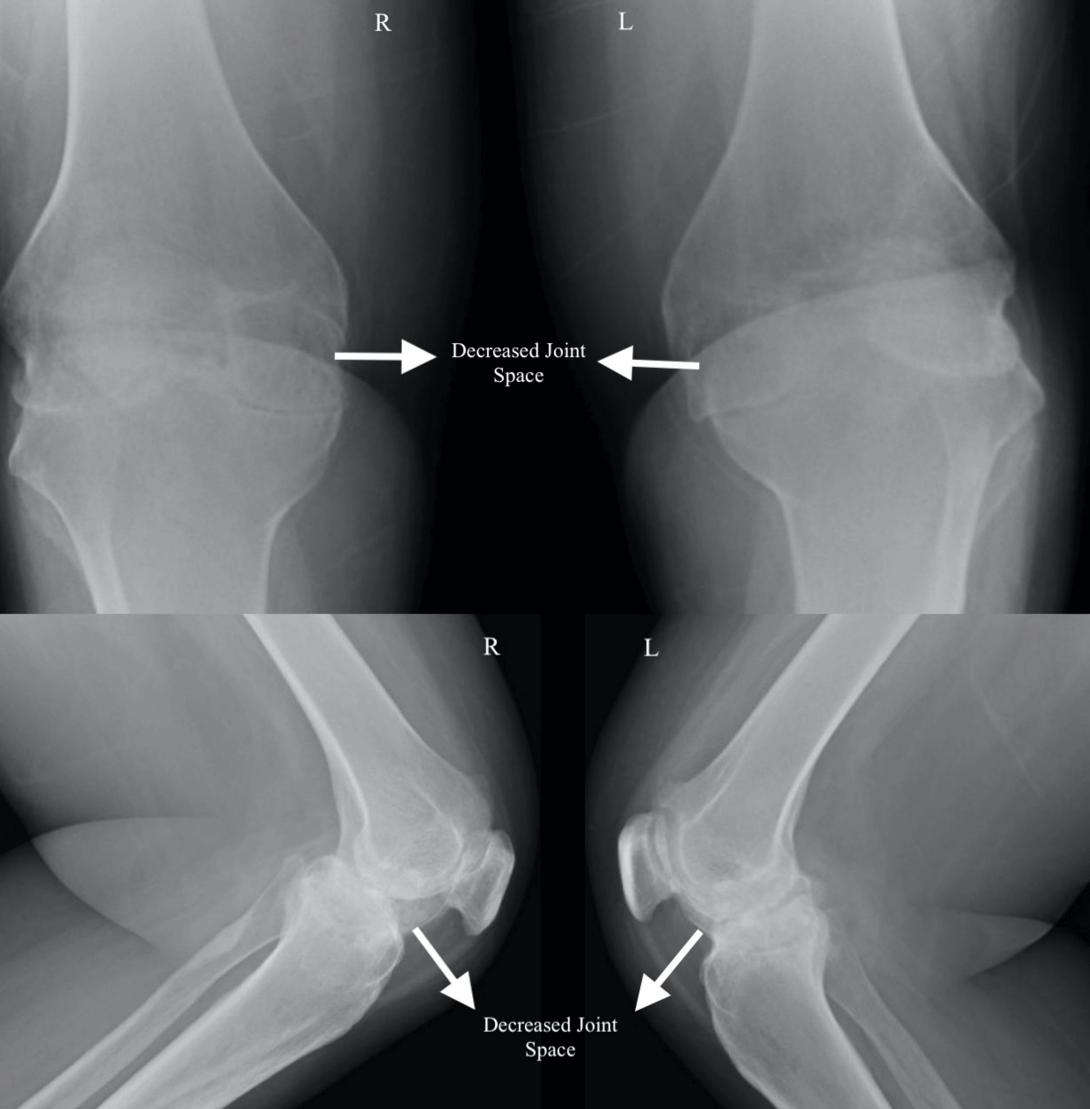
Anteroposterior and lateral X-rays showing severe osteoarthritis of both knees with decreased joint space

A 73-year-old female diagnosed with bilateral OA knees was scheduled to undergo bilateral TKR. The severity of the OA (as seen in Figure 1) had progressed to a level where she experienced significant pain and limited mobility, making surgery a necessary course of action. The patient's medical history included a prior bilateral tibial fracture, for which a combined spinal epidural anaesthesia technique was intended. Due to complications when placing the epidural catheter, the tibial fracture procedure was carried out under general anaesthesia.

Pre-anaesthetic evaluation

**Figure 2:**
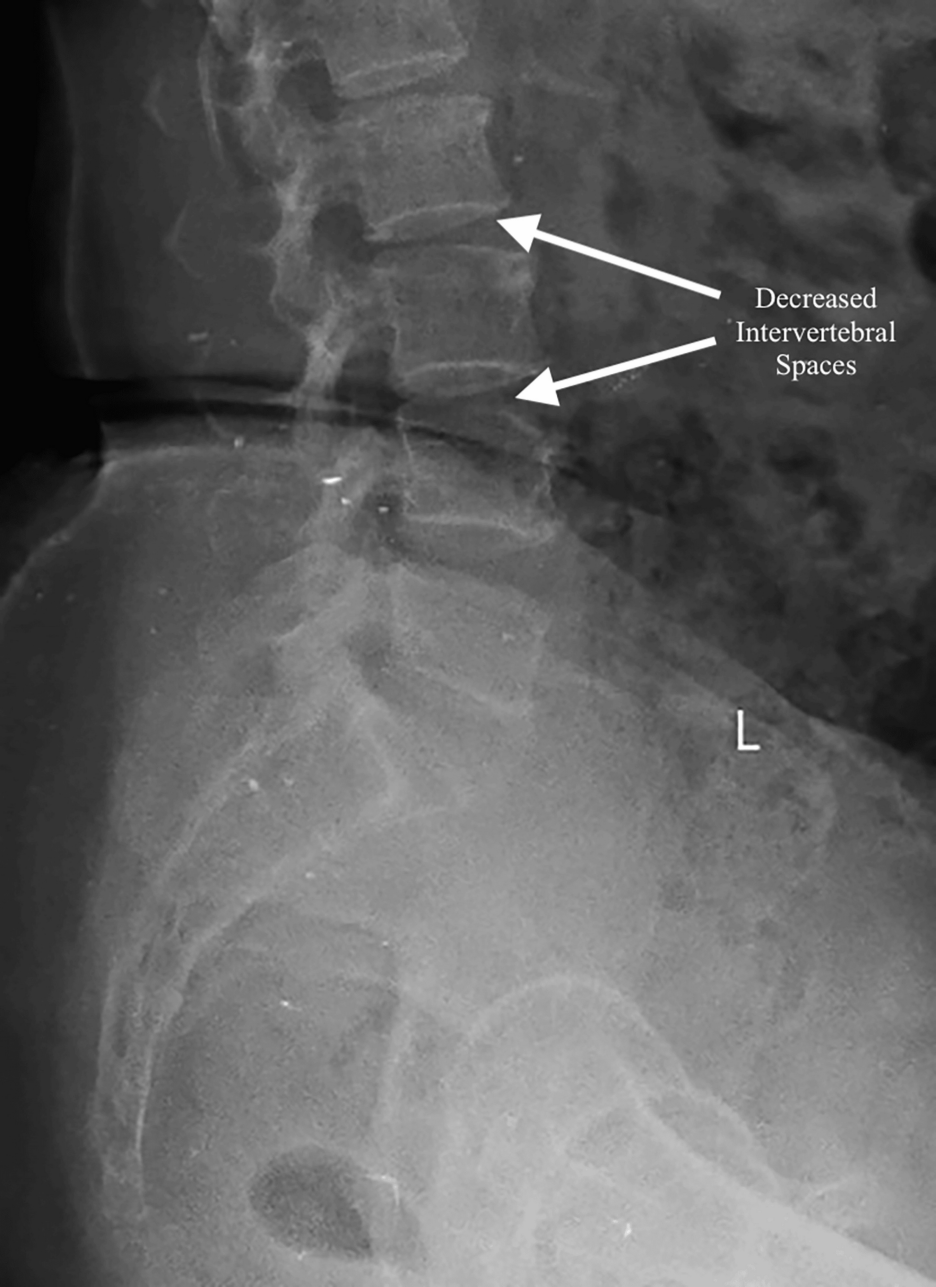
Xray of the lumbar spine showing decreased intervertebral spaces

The patient complained of pain in both knees with stiffness and a decrease in functional capacity. The BMI of the patient was noted to be 27.2 kg/m². The patient had a history of bilateral OA knee, which was treated with medication and physiotherapy. On the contrary, no significant comorbid conditions were found, and systemic examination findings were also within normal limits. She had one previous surgery for a bilateral tibial fracture seven years ago, where the planned epidural and spinal anaesthesia had to be abandoned and general anaesthesia was given because of difficulty in placing the epidural catheter. No functional cardiac incapacitation to surgery was noted on preanesthetic examination. Pulse rate was 80/minute, blood pressure was 120/80 mmHg, respiratory rate was 16 breaths/minute, and oxygen saturation (SpO2) was 98% on room air. All routine laboratory investigations like complete blood count, renal function tests, and liver function tests were within normal limits (Table 1). The preceding anaesthetic history necessitated an X-ray examination of the lumbar spine, which revealed significant degenerative changes and reduced intervertebral spaces indicative of the ageing process (Figure 2). Given the patient's previous difficulties with epidural insertion and the radiographic findings, it was decided to do a bilateral TKR and to employ ultrasound guidance for the epidural catheterisation, with general anaesthesia as a contingency. Following a detailed explanation of the risks and advantages associated with the procedure, informed consent was obtained.

**Table 1:** Laboratory values

Laboratory Test	Result	Reference Range
Complete Blood Count (CBC)		
Hemoglobin (g/dL)	13.5	12.0 - 15.5 (Female)
Hematocrit (%)	40	37.0 - 47.0 (Female)
White Blood Cell Count (×10³/µL)	6.5	4.0 - 10.0
Platelet Count (×10³/µL)	250	150 - 450
Renal Function Tests		
Serum Creatinine (mg/dL)	0.9	0.6 - 1.2
Blood Urea Nitrogen (mg/dL)	15	7-20
Liver Function Tests		
Alanine Aminotransferase (U/L)	20	7-56
Aspartate Aminotransferase (U/L)	18	10-40
Alkaline Phosphatase (U/L)	70	44 - 147
Total Bilirubin (mg/dL)	0.8	0.1 - 1.2

Anaesthetic management

**Figure 3:**
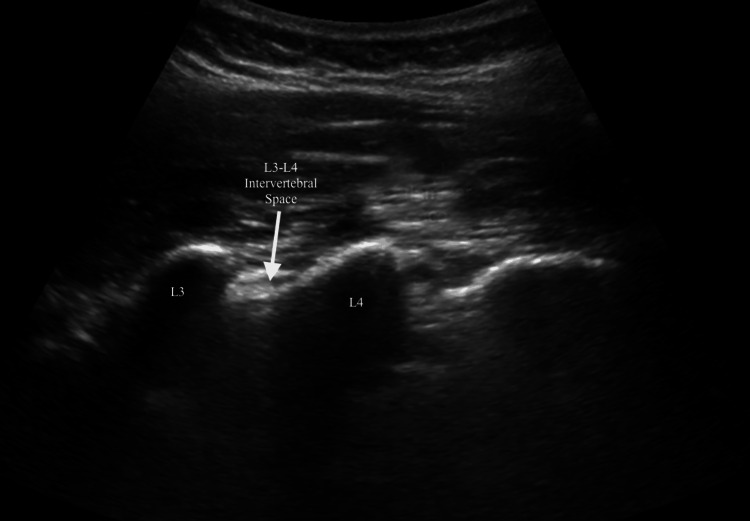
Ultrasound image showing L3-L4 intervertebral space

An 18G intravenous access was established during arrival in the operation theatre and fluid therapy was instituted to ensure adequate hydration with hemodynamic stability. The monitors attached included electrocardiography, pulse oximetry, and a non-invasive blood pressure and heart rate monitor. Base-line parameters were pulse rate of 78 bpm, blood pressure of 118/76 mmHg, respiration rate of 16 breaths/minute, and SpO2 of 100% on room air, which indicated that the patient was in stable preoperative condition. The patient was positioned in the sitting position to allow for the precision of the neuraxial block and to maximize access to the lumbar spine for the anesthesiologist. The position enhanced accuracy in the placement of the needle by allowing for the correct orientation of the spinal structures. Ultrasound guidance was utilized to perform spinal and epidural anesthesia techniques as seen in Figure 3.

The posterior epidural space was identified, followed by the visualization of the lumbar spine with a linear probe operating within a frequency range of 3-13 MHz. Keeping the orientation marker cranially, the probe was placed in the paramedian sagittal plane, approximately 1-2 cm lateral to the spinous processes. This technique allowed visualization of the L3-L4 intervertebral space, the intended target site for the epidural block. An in-plane approach was used with the insertion of the 18-gauge Tuohy needle from the caudal direction, with its advancement watched in real time under ultrasound guidance as it moved toward the ligamentum flavum and into the epidural space. This helped minimize the complication rate while allowing correct placement of the needle. A catheter was then introduced through the needle and placed in the epidural space following confirmation of appropriate placement by loss of resistance technique. After that, a sterile dressing was used to secure the catheter. A 25G Quincke needle was used to inject a combination of 15 mg of hyperbaric bupivacaine and 5 mcg of dexmedetomidine into the subarachnoid space at the L3-L4 intervertebral space, following an identical approach to spinal anaesthesia with ultrasonographic guidance. Dexmedetomidine was given to improve the analgesic and sedative effects of the spinal block and to extend its duration. A test dose of 3 mL of 1.5% lidocaine with epinephrine was given through the epidural catheter to verify proper catheter placement and guarantee safety. Supplemental dosages of 10 ml of 0.25% bupivacaine were given every 45-60 minutes to make certain that there was satisfactory analgesia during the time of surgery, and no other intravenous analgesic was given.

Surgical management

**Figure 4:**
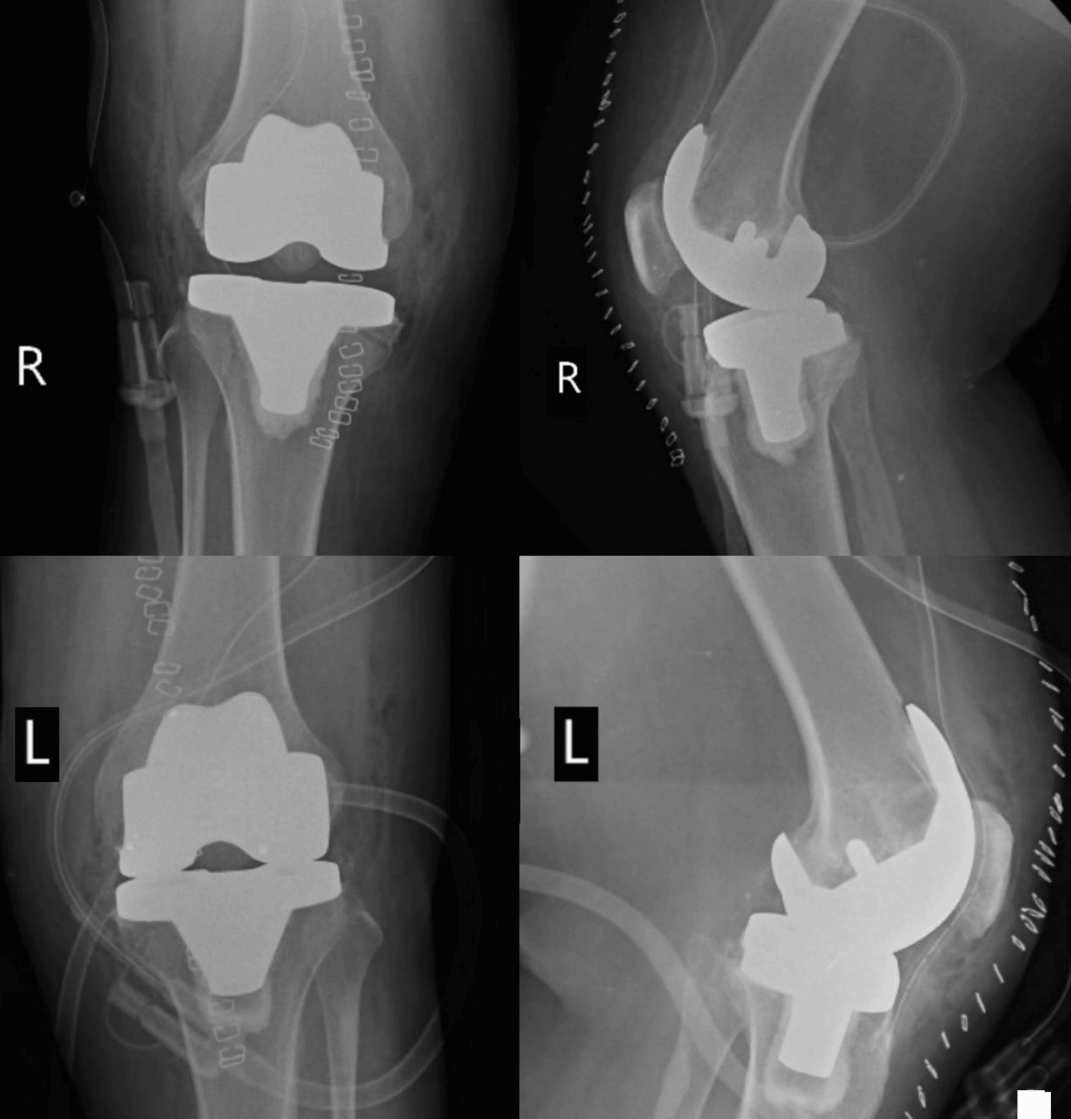
Postoperative X-ray of both knees showing implants

Bilateral medial parapatellar incisions were made. Much care was taken at each incision to make sure of minimum trauma to the soft tissues. The soft tissues of both knee joints were carefully dissected to expose the knee joints. The patellae were mobilized and retracted to give good exposure to the femur and tibia. Subsequently, resection started after the knees had been exposed, including specified bone cuts to prepare the surface for total knee implants, also aligning them accordingly with adequate preparation of the bony surfaces following meticulous removal of the degenerated bone and osteophytes in both knees from the femoral and tibial surfaces. Following this, the whole knee prostheses for both knees were precisely prepared and positioned. Each knee's femoral and tibial components were implanted following the guidelines provided by the manufacturer (Meril Life Sciences Pvt. Ltd., Gujarat, India). To get the best possible joint function and stability, it was made sure the implants were securely fixed and aligned. Each knee joint was extensively irrigated to guarantee haemostasis and eliminate any debris following the implants. Absorbable sutures were used to close the wounds in layers, and sterile bandages were placed on each knee to aid in healing and preserve the surgical sites. Postoperative X showing the implants are seen in Figure 4.

Postoperative management

The patient was kept in the postoperative unit for four hours and then shifted to the ward. During the postoperative stage, continuous epidural analgesia was continued with 10 ml of 0.125% bupivacaine administered every six to eight hours through the catheter for 48 hours. This regimen provided excellent pain control with reduced consumption of systemic analgesics. She was discharged on postoperative day three after a satisfactory recovery with appropriate follow-up instructions and oral analgesics.

## Discussion

OA of the knee is widespread, especially in older patients. It may cause severe pain coupled with poor mobility which will raise the need for TKR [1]. Under such circumstances, as reported here, many challenges arise in anaesthesia management in such patients presenting with severe OA and a history of difficult epidural placements. Besides that, anatomical changes due to age and complications from previous surgeries may render conventional anesthetic techniques ineffective. The case highlighted the difficulties with epidural anaesthesia due to marked degenerative spinal changes, having had tibial fracture repair where epidural placement was difficult, and hence was done under general anaesthesia. The preoperative imaging of the lumbar spine showed significant degenerative changes with decreased intervertebral spaces that could distort the anatomical landmarks and complicate the identification and placement of an epidural catheter; thus, it is necessary to tailor anaesthetic strategies based on the scenario. Most conventional approaches to epidural anaesthesia rely on anatomical landmarks and palpation, which, in turn, are highly unreliable in patients with such pronounced degenerative changes in the spine.

Anatomical distortion and reduced intervertebral space can make it challenging to locate the epidural space precisely, which increases the risk of multiple needle attempts and procedural discomfort. These methods also increase the procedural difficulty and the risk of complications, such as accidental dural puncture or inadequate analgesia [5]. Ultrasound-guided neuraxial anaesthesia is an advancement over conventional techniques as it provides real-time visualization of the spinal anatomy. This allows better localization of the epidural space, thus more precise placement of the needle, reducing the possibility of complications arising and hence increasing the success rate of the procedure. In the current case, ultrasound guidance played a vital role in overcoming anatomical difficulties due to the patient's degenerative changes. Linear probes provide high-resolution images within a frequency range of 3-13 MHz of the lumbar spine and hence allow for accurate and precise localization of intervertebral spaces, which are important during the administration of epidural and spinal anaesthesia [6].

Ultrasound guidance increased overall procedural safety while at the same time augmenting precision in needle placement. With the capability to visualize the path of the needle in real time, the number of insertions could be reduced, thereby minimizing discomfort for the patient. This approach helped to execute successful insertions of both the spinal needle and the placement of an epidural catheter important for perioperative pain management. Other factors contributing to patient comfort, other than the use of ultrasound guidance in the procedure, were the addition of dexmedetomidine to the formulation of spinal anesthetic. Dexmedetomidine has been reported as exhibiting properties contributing to analgesia, extending the quality and duration of the spinal block, offering additional analgesia, and maintaining intraoperative stability [7]. Epidural boluses of bupivacaine allowed adequate analgesia in the postoperative period, with less systemic use of analgesia, and were given every six to eight hours through the epidural catheter. This kept consistent analgesia throughout the recovery period. The patient's discharge on the third postoperative day with oral analgesics demonstrated the effectiveness of the perioperative pain management plan.

## Conclusions

This case report outlines the fact that the use of ultrasound guidance while carrying out neuraxial anaesthesia does confer very significant benefits in both anatomically difficult patients and those with a history of difficult epidural placements. It enhances the preciseness of needle placement, thereby enhancing pain management in a way that the more significant part of inefficiencies gets neutralized. This ensures that in the constantly evolving field of anesthesia, the usage of ultrasound improves patient outcomes, particularly those with very challenging anatomy and complex anaesthesia requirements.
